# Automatically detecting Crohn’s disease and Ulcerative Colitis from endoscopic imaging

**DOI:** 10.1186/s12911-022-02043-w

**Published:** 2022-11-18

**Authors:** Marco Chierici, Nicolae Puica, Matteo Pozzi, Antonello Capistrano, Marcello Dorian Donzella, Antonio Colangelo, Venet Osmani, Giuseppe Jurman

**Affiliations:** 1 Fondazione Bruno Kessler, via Sommarive, 18, 38123 Trento, Italy; 2 PagoPA S.p.A., Rome, Italy; 3 Università degli studi di Trento, via Calepina, 14, 38122 Trento, Italy; 4 GPI S.p.A., via Ragazzi del ’99, 13, 38123 Trento, Italy

**Keywords:** Artificial intelligence, Machine learning, Inflammatory bowel disease, Endoscopy, Predictive models, Diagnosis, Ulcerative Colitis, Crohn’s disease

## Abstract

**Background:**

The SI-CURA project (*Soluzioni Innovative per la gestione del paziente e il follow up terapeutico della Colite UlceRosA*) is an Italian initiative aimed at the development of artificial intelligence solutions to discriminate pathologies of different nature, including inflammatory bowel disease (IBD), namely Ulcerative Colitis (UC) and Crohn’s disease (CD), based on endoscopic imaging of patients (P) and healthy controls (N).

**Methods:**

In this study we develop a deep learning (DL) prototype to identify disease patterns through three binary classification tasks, namely (1) discriminating positive (pathological) samples from negative (healthy) samples (P vs N); (2) discrimination between Ulcerative Colitis and Crohn’s Disease samples (UC vs CD) and, (3) discrimination between Ulcerative Colitis and negative (healthy) samples (UC vs N).

**Results:**

The model derived from our approach achieves a high performance of Matthews correlation coefficient (MCC) > 0.9 on the test set for *P versus N* and *UC versus N*, and MCC > 0.6 on the test set for *UC versus CD*.

**Conclusion:**

Our DL model effectively discriminates between pathological and negative samples, as well as between IBD subgroups, providing further evidence of its potential as a decision support tool for endoscopy-based diagnosis.

## Introduction

Inflammatory bowel diseases (IBD), including Crohn’s disease (CD) and Ulcerative colitis (UC), are chronic and recurrent diseases. CD patients have healthy parts of the intestine mixed in between inflamed areas, while UC induces a continuous inflammation of the colon. Further, CD may occur in all the layers of the bowel walls, while UC only affects the innermost lining of the colon. Although they both have an undetermined etiology, research advances have outlined some of the pathways leading to their insurgence: (1) genetic predisposition associated with the environment induces disruption of the intestinal microbial flora; (2) the structure of the epithelial cells and of the immune system of the intestine determine the risk of developing the disease. IBD is diagnosed using a combination of endoscopy (for CD) or colonoscopy (for UC) and imaging studies, such as contrast radiography, magnetic resonance imaging, or computed tomography. Physicians may also check stool samples to make sure symptoms are not being caused by an infection or run blood tests to help confirm the diagnosis. Still, a definite diagnosis of IBD remains a challenging task [1], often affected by subjective judgement [2]. Several automated approaches have been published in the recent literature [2, 3] attempting to provide computational support to improve the diagnostic task, with machine learning (ML) models playing a major role, however several challenges remain [4, 5]. In this line Stidham et al. [6] found deep learning model performance to be similar to an experienced clinician in evaluating severity of UC, while Takenaka et al. [7] derived a high performance model in identifying UC patients with endoscopic remission. Klein et al. [8] focused on analysis of colonic biopsies of CD patients to predict post-biopsy clinical phenotypes and outcomes, similar to Waljee et al. [9, 10] developing a model to predict IBD-related hospital admission and disease flare.

As a consequence, the emergence of Artificial Intelligence (AI) solutions based on Deep Learning (DL) models comes as no surprise. The models exploit the potential of artificial neural networks to automatically process different types of data [11, 12], including endoscopic imaging [13]. Our work naturally embeds in this research line, combining recent DL architectures with more classical ML strategies such as ensemble learning to further enhance the predictive performance. The promising results obtained can support the clinicians in providing a more objective and reliable diagnosis, thus reducing the risk of misidentification of CD and UC, an important aspect considering different treatment options and follow-ups of the two conditions [13].

## Methods

**Fig. 1:**
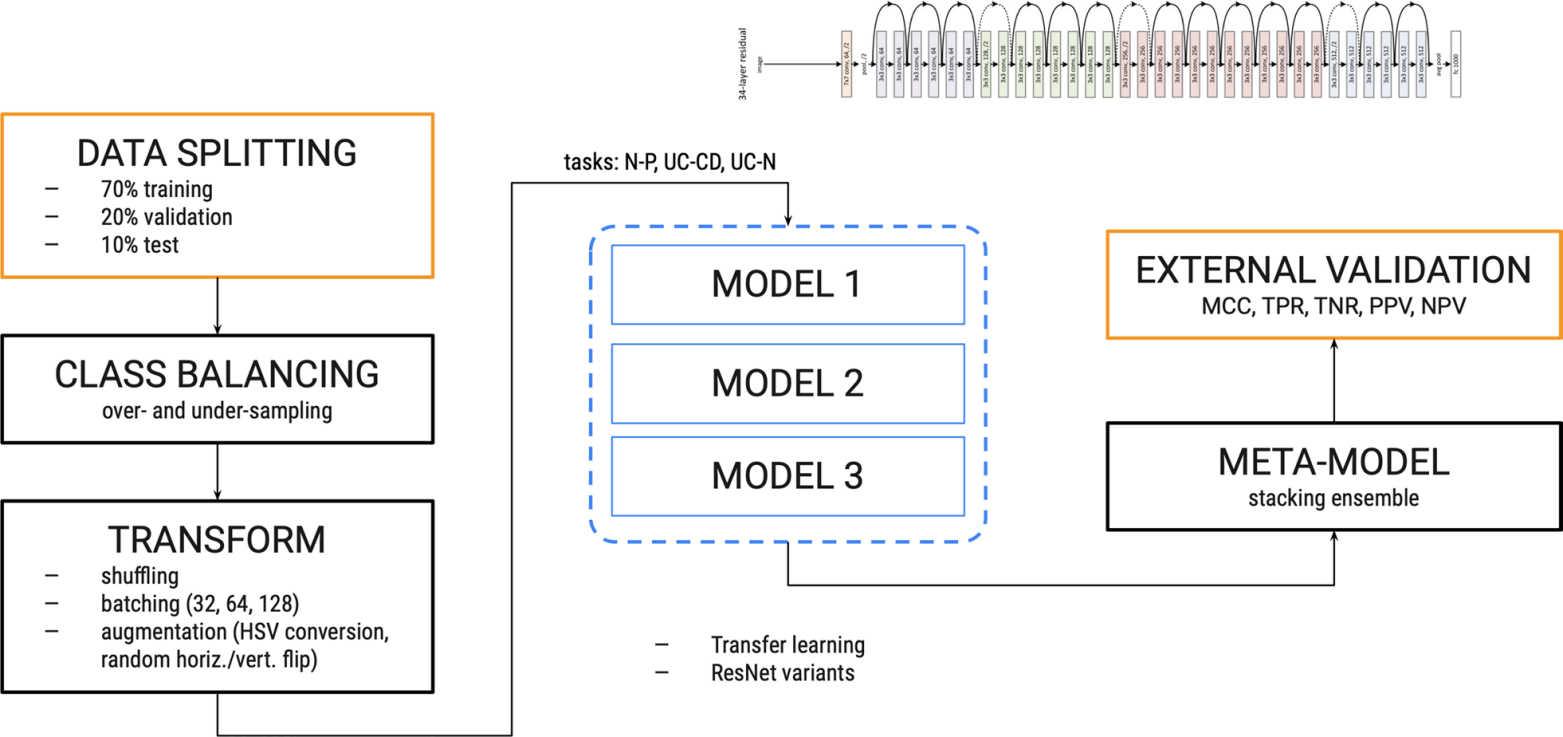
Experimental workflow. After an upstream processing of the input images (blocks “Data splitting”, “Class balancing”, “Transform”), for each classification task three pre-trained ResNet variants of increasing complexity (“Model *i*”, $i = 1,2,3$) are used as weak learners and fine-tuned in 5-fold cross-validation on the input data (see Methods). A meta-model is then built by stacking ensemble of the three weak learners, evaluating the performance on the external test set in terms of different classification metrics (see Methods). N–P, negative versus positive; UC–CD, Ulcerative Colitis versus Crohn’s Disease; UC–N, Ulcerative Colitis versus negative; MCC, Matthews Correlation Coefficient (MCC); TNR, true negative rate; TPR, true positive rate; NPV, negative predictive value; PPV, positive predictive value

We tackle the problem of IBD detection from endoscopic imaging with an ensemble learning approach based on a fine-tuned ResNet architecture. An overview of the general workflow is presented in Fig. 1. Briefly, the input images first undergo a random partitioning into train and test sets: the train set is used for model development in a k-fold cross-validation (CV) schema, while the test set is kept apart to obtain predictions from the trained model. Then, after a preprocessing phase aimed at preparing the images for the predictive modeling, three pre-trained Residual Network models [14] of increasing complexity are fine-tuned on the preprocessed images in a transfer learning setting. Finally, the models are combined in a single meta-model with a stacking ensemble method.

### Outcome definition

In this work, we investigate three major outcomes, namely: (1) discrimination between negative (healthy) samples and positive (pathological) samples (N–P), (2) discrimination between Ulcerative Colitis and Crohn’s Disease (UC–CD) samples and, (3) discrimination between negative samples and Ulcerative Colitis (UC–N).

### Dataset description

**Fig. 2:**
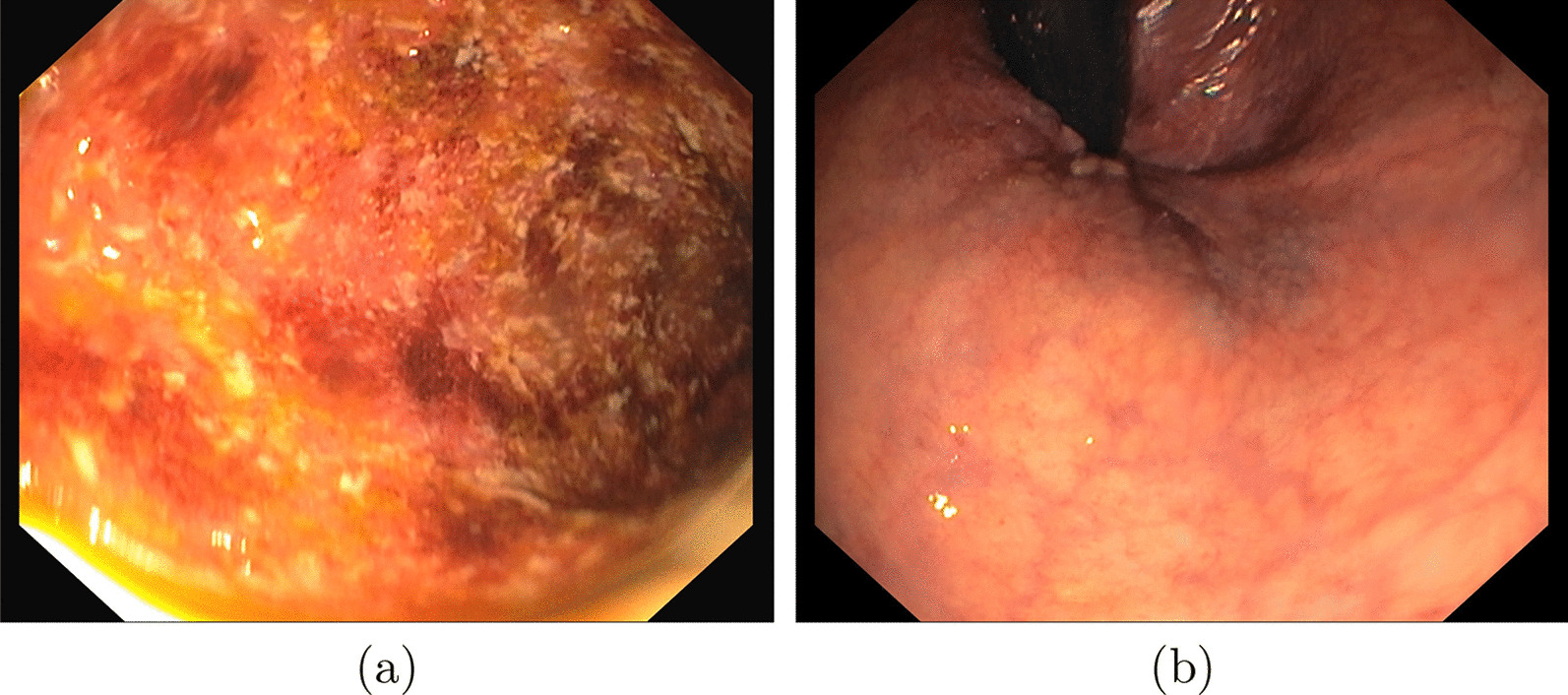
An example of positive (**a**) and negative (**b**) images within the SI-CURA dataset

The SI-CURA dataset includes 14,226 three-channel RGB (red, green, blue) endoscopic images of different sizes split between “Positive” patients (P, n = 11,404) and “Negative” healthy controls (N, n = 2822). In this work, we consider a single image to be a sample for the classification task. Positive (pathological) samples are further labeled as Ulcerative Colitis (UC), Crohn’s disease (CD), and Inflammatory bowel disease (IBD). Table 1 illustrates the sample stratification for each class and Fig. 2 shows an example of positive and negative images.

In developing and evaluating the model, the overall dataset was split into two main subsets: training (90% of total sample size) and test (10%). The training set was further split for model development according to a 5-fold cross-validation schema. Since the P data is 3$\times$ the N data, under- and over-sampling were used to balance the class distributions using the Imbalanced-dataset-sampler Python library,^1^ which automatically estimates the sampling weights and mitigates overfitting when used in conjunction with data augmentation techniques.

### Data preprocessing

An image editor was used to remove undesired artifacts, such as signs, writings, medical instruments, black, white, and corrupted images. Further, all images were converted to JPEG with lossy compression to have a consistent format across the dataset. Table 1 details the number of elements in each class after preprocessing.

Data augmentation is a technique commonly used to increase the amount of relevant data in the original dataset, thus providing additional examples to the neural network. Augmentation exploits the fact that convolutional neural networks are invariant to transformations such as translation, size, viewpoint, rotation. The following techniques were used: RGB to HSV (hue, saturation, value) colour model conversion, random horizontal flip, and random vertical flip, all of which had an equal probability of being applied.

Several experiments were performed with different batch and image sizes. The best results were obtained with a batch size of 32, 64, and 128, depending on the model’s depth. To speed up the training time, we rescaled all images to 700$\times$700 pixels, equal to the average image dimension.

### Deep learning architecture

We tackled the tasks through a transfer learning approach using the following variants of Residual Networks (ResNet) [14]: ResNet18, ResNet34, ResNet50, ResNet101, and ResNet152, all of which were imported pre-trained on ImageNet from the PyTorch Hub.^2^ The choice of using pre-trained models leverages the information already learned by the network after an extensive training on the large ImageNet dataset. The fully-connected head of the ResNet networks was swapped with one performing binary classification: an untrained sequential module ending with a linear transformation and two outputs. Moreover, the rectified linear unit function (ReLU) [15] and dropout technique (with $p = 0.5$ probability) were used in this module. ReLU activation has been widely used in deep neural networks due to its advantages over other functions (e.g. sigmoid): most importantly, it overcomes the vanishing gradient problem [16] that has been afflicting neural networks for several years. We adopted dropout to increase regularisation, preventing the coadaptation of the neurons. The weights of the layers were frozen except for the last two, which were updated with a small variation of the learning rate. In a typical transfer learning approach, the layers of the network are frozen to the trained weights except for the last (“higher”, or “top”) layers (farther to the input). This is because the first-layer features are more general and the last-layer features are more specific [17]. There are different strategies for fine-tuning the higher layers: typically, the final fully-connected classification part of the network is trained, leaving the convolutional layers frozen. However, it is also possible to unfreeze one or more convolutional layers as well to fine-tune the performance. We chose to unfreeze the last two convolutional layers as a compromise between achieving a good performance and avoiding overfitting, following recommendations in the literature [18]. The optimiser used for the training was Adaptive Movement Estimation (Adam) [19], a replacement for Stochastic Gradient Descent that combines the advantages of the Adaptive Gradient (AdaGrad) and Root Mean Square Propagation (RMSProp) algorithms, making it a popular choice in recent studies [20]: AdaGrad maintains a per-parameter learning rate that improves performance on problems with sparse gradients, and RMSProp also maintains per-parameter learning rates using a decaying average of recent partial gradients. This means that Adam improves parameter optimisation, particularly in online and non-stationary (e.g., noisy) tasks. The actual number of epochs was determined by early stopping based on the validation loss, with a patience of 30 epochs. The maximum number of epochs was set to 200, a value high enough to allow the net to learn as much as possible until the early stopping technique takes place.

### Differential learning rates

The learning rate is one of the most important hyperparameters that can be tuned to improve optimisation convergence. It is usually chosen by time-consuming techniques such as grid search that exhaustively experiments with different values, picking the one that works best in terms of performance on the validation set. A more convenient approach, which was adopted in this study, is the learning rate finder [21]. During each epoch, the algorithm makes the learning rate cyclically vary between a lower and an upper bound. The loss corresponding to each learning rate is computed, and the learning rate yielding the steepest drop in the loss is chosen.

The learning rate finder method was complemented by the use of different learning rates during the training, an approach commonly referred to as “differential learning rates”, which is particularly useful in a transfer learning setting. In fact, only the layers immediately preceding the fully-connected classifier head were unfrozen and trained. Since the ResNet model is pre-trained on the large ImageNet dataset, these layers only need a small amount of fine-tuning to achieve good prediction performance. In particular, the learning rate values for the ResNet’s layer4 (just before the classifier) and layer3 were reduced by a third and by a ninth, respectively.

### Ensemble learning

Ensemble learning is a machine learning paradigm where multiple models (“weak learners”) are trained to solve the same problem and finally combined to achieve better results. Two ensemble methods were used in this study: averaging prediction and stacking. While the former keeps the models independent from each other and averages their predictions, the latter combines the predictions of the weak learners through a final classifier, resulting in a meta-model. We combined three weak learners in a stacking ensemble meta-model: ResNet34, ResNet50, and ResNet101 were used as weak learners in the N–P and UC–N cases, and ResNet34, ResNet50, and ResNet152 in the UC–CD case. Other combinations were tried, but they performed worse. The ensemble meta-model has six input features (two for each weak learner) and two outputs features, and it was further trained for several epochs by using the early stopping criterion.

### Performance evaluation metrics

The metrics used for assessing the model performance in both training and evaluation stages are Matthews Correlation Coefficient (MCC), true positive rate (TPR), true negative rate (TNR), positive predictive value (PPV), negative predictive value (NPV), and the confusion matrices. The MCC was used as the main metric because it is particularly suitable for binary and multiclass classification [22], and it is generally regarded as a balanced performance measure that can be used even if the classes are of very different sizes. MCC is computed from the values of the confusion matrix, true positives (TP), true negatives (TN), false positives (FP), and false negatives (FN), as in the following:

$$\begin{aligned} \textrm{MCC}=\frac{\textrm{TP}\times \textrm{TN}-\textrm{FP}\times \textrm{FN}}{\sqrt{(\textrm{TP}+\textrm{FP})(\textrm{TP}+\textrm{FN})(\textrm{TN}+\textrm{FP})(\textrm{TN}+\textrm{FN})}} \end{aligned}$$

The values of MCC range between −1 and 1, where 1 means perfect classification, −1 perfect misclassification (inverse prediction), and 0 random guess or, generally speaking, absence of correlation between the predictions and the ground truth.

We also computed the training and validation losses and the 95% studentized bootstrap confidence intervals for the training MCC.

### Model interpretation

In an attempt to better understand which input features are deemed important by the trained deep learning models, we used two explainable AI (XAI) algorithms implemented in the Captum library,^3^ namely Saliency and Guided backpropagation. Saliency [23] is one of the most straightforward approaches for estimating input attribution: it computes the gradient of the model output with respect to the input pixels of a given image. While a simple approach to deep model interpretation, Saliency maps cannot capture the input feature interactions and tend to be noisy. Guided backpropagation [24] (GBP) is an extension of the Saliency algorithm aiming to alleviate these issues: GBP also computes the gradient of the output with respect to the input; in addition, it overrides ReLU backpropagation, resulting in backpropagation of non-negative gradients only. Each method computes an attribution value (the higher, the more important) for each input pixel: the resulting attribution maps are then qualitatively assessed by overlaying them on the original image.

### Implementation

The following frameworks and libraries were used: PyTorch v1.5.0 with torchvision v0.6.0a0, Captum v0.4.1, CUDA 10.2, and Jupyter v6.1.4. Color model conversion was performed by the PIL library. The packages were installed in a Anaconda virtual environment for improved reproducibility. The analyses were run on a ppc64le server, provided by GPI S.p.A., with 128 CPUs (max 4023 MHz, min 2394 MHz) and 4 NVIDIA Tesla P100 SXM2 16GB GPUs. The training of neural networks was performed on multiple GPUs using PyTorch’s DataParallel.

## Results

### Classification

**Fig. 3:**
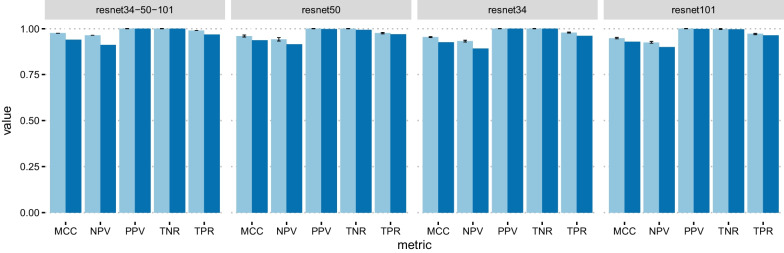
N–P classification results. Metrics values for the ensemble model (resnet34-50-101) and the weak learners resnet34, resnet50, and resnet101. Light blue: cross-validation; dark blue: test set. Black bars represent 95% confidence intervals

**Fig. 4:**
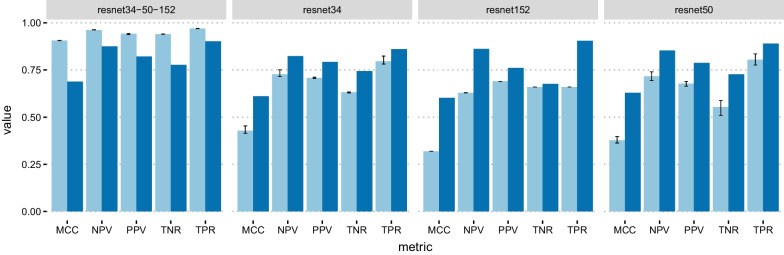
UC–CD classification results. Metrics values for the ensemble model (resnet34-50-152) and the weak learners resnet34, resnet50, and resnet152. Light blue: cross-validation; dark blue: test set. Black bars represent 95% confidence intervals

**Fig. 5:**
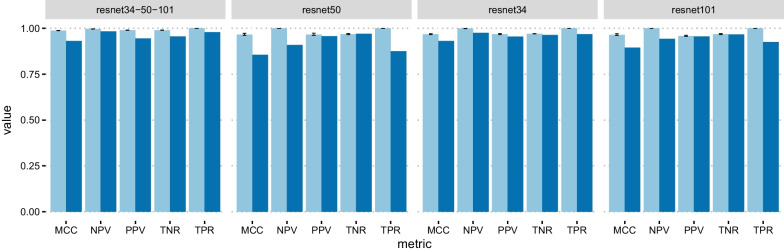
UC–N classification results. Metrics values for the ensemble model (resnet34-50-101) and the weak learners resnet34, resnet50, and resnet101. Light blue: cross-validation; dark blue: test set. Black bars represent 95% confidence intervals

**Fig. 6:**
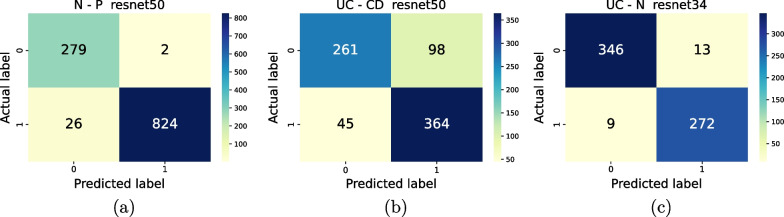
Confusion matrices. Test set confusion matrices for the best-performing weak learners of each classification task (see Table 2)

Figures 3, 4, 5 show the cross-validation and test set performance of the weak learners and the ensemble meta-models for all classification tasks. The test set confusion matrices are reported in Fig. 6 for the best-performing weak learner of each task. Table 2 reports the results in terms of average cross-validation MCC with 95% confidence intervals and the test set MCC, TPR, TNR, PPV, and NPV. The rows of Table 2 are ranked task-wise by decreasing test set MCC. The average-prediction ensemble models performed consistently worse for all tasks and are not shown. According to Table 2 and Fig. 3, the top three nets (or weak learners) for N–P are ResNet50, ResNet34, and ResNet101. While the first one is trained with an unbalanced training data loader, the latter two nets are trained using a balanced data loader.

**Table 1:** Number of elements (images) in each class, in the format “raw data (preprocessed data)”

P			N
UC	CD	IBD	
4388 (3594)	5949 (4098)	1067 (823)	2822 (2815)

*P*, positive; *N*, negative; *UC*, Ulcerative Colitis; *CD*, Crohn’s disease; *IBD*, inflammatory bowel disease

**Table 2:** Metrics values for the best trained nets, ranked by decreasing Matthews Correlation Coefficient (MCC) on the test set; cv, ts: cross-validation and test set; TNR, true negative rate; TPR: true positive rate; NPV, negative predictive value; PPV, positive predictive value; resnetX-Y-Z: meta-model obtained by stacking ensemble of the three weak learners resnetX, resnetY, and resnetZ

Task	Net	*MCC*_*cv*_	*MCC*_*ts*_	*TNR*_*ts*_	*TPR*_*ts*_	*NPV*_*ts*_	*PPV*_*ts*_
N–P	resnet34-50-101	0.976 (0.976,0.976)	0.940	1.000	0.968	0.912	1.000
	resnet50	0.960 (0.954,0.966)	0.937	0.993	0.969	0.915	0.998
	resnet101	0.948 (0.944,0.952)	0.929	0.996	0.964	0.900	0.999
	resnet34	0.954 (0.951,0.956)	0.925	1.000	0.960	0.892	1.000
UC–CD	resnet34-50-152	0.906 (0.905,0.907)	0.688	0.777	0.902	0.875	0.822
	resnet50	0.377 (0.362,0.398)	0.629	0.727	0.890	0.853	0.788
	resnet34	0.428 (0.415,0.454)	0.611	0.744	0.861	0.824	0.793
	resnet152	0.319 (0.319,0.319)	0.602	0.677	0.905	0.862	0.761
UC–N	resnet34-50-101	0.988 (0.988,0.988)	0.931	0.955	0.979	0.983	0.945
	resnet34	0.968 (0.966,0.970)	0.930	0.964	0.968	0.975	0.954
	resnet101	0.964 (0.961,0.970)	0.895	0.967	0.925	0.943	0.956
	resnet50	0.966 (0.962,0.972)	0.855	0.969	0.875	0.909	0.957

As for the UC–CD task, the best results were obtained by ResNet34, ResNet50, and ResNet152 (Table 2, Fig. 4). The first two nets were trained with a balanced data loader and the last one with an unbalanced data loader. In this task, the classes are not as imbalanced as in N–P (Table 1): however, the dataset balancing technique helped maintain balanced the confusion matrices in the training and evaluation phase. As expected, the meta-model slightly improves over the weak learners on the test set (Table 2).

The best-performing networks for the UC–N task are a ResNet34 model with a balanced data loader and an ensemble model using ResNet50, ResNet34, and ResNet101 as weak learners trained with unbalanced, balanced, and unbalanced data loaders, respectively. The best metrics are shown in Table 2. In this case, the performance improvement of the ensemble model over the single best-performing weak learner ResNet34 is limited.

The performance on the test set is reasonably comparable to that in cross-validation for all tasks and models, except for the UC–CD ensemble model which exhibits higher CV performance (MCC = 0.906 vs. 0.688, Fig. 4, Table 2).

### Interpretability

**Fig. 7:**
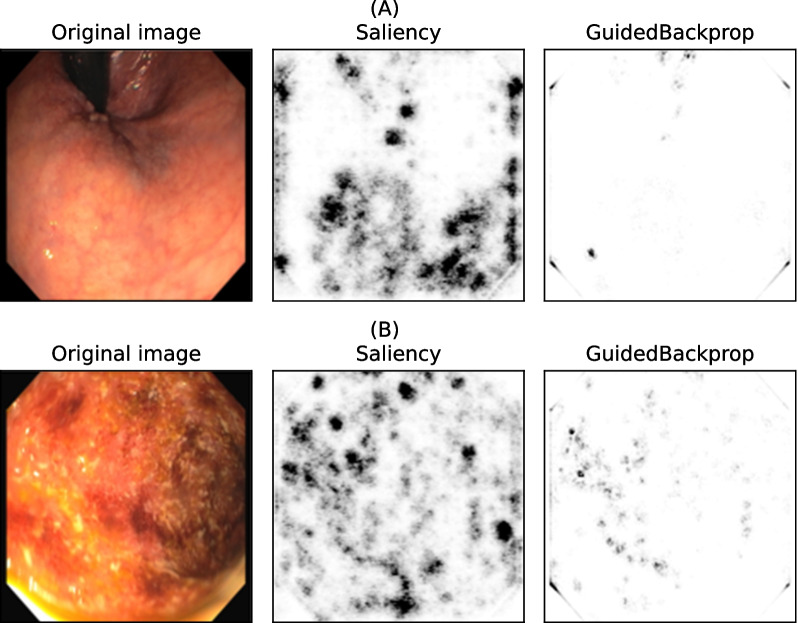
Model interpretability. Qualitative visualization of attribution maps by Saliency and Guided backpropagation (GuidedBackProp) algorithms for a Negative (**A**, top row) and Positive (**B**, bottom row) input image. The original image is also shown for comparison. The attribution maps are visualized as heatmaps, with darker shades representing more important features according to the algorithm

We applied the chosen model interpretation methods to a random subsample of test set N–P images using a ResNet50 model, which was the best performing weak learner on the task N–P (Table 2). For two representative images (1 Positive, 1 Negative), Fig. 7 visualizes the Saliency and Guided Backpropagation (GuidedBackProp) attribution maps overlaid on the original image, which is also shown separately for comparison. We observe that GuidedBackProp attributions are less noisy than Saliency ones, as expected. Moreover, typical endoscopic features of IBD such as mucosal erythemas appear to have higher attribution values according to GuidedBackProp (Fig. 7, bottom). Using a gradient-based XAI method such as Saliency and Guided backpropagation is particularly appealing because it allows a straightforward visual interpretation of the input features deemed important by the underlying model. However, the identified features should be the object of further analyses since they may be affected by different kinds of bias, such as illumination (Fig. 7).

## Discussion

In this work, we developed and evaluated a prototype DL framework based on ResNet architectures merged by ensemble learning, able to identify disease patterns from endoscopic images of different IBDs, namely Ulcerative Colitis and Crohn’s Disease, and distinguish negative (healthy) samples. The DL models achieve a test set performance MCC = 0.940 for the classification of healthy controls versus IBD patients, MCC = 0.688 for Ulcerative Colitis versus Crohn’s Disease, and MCC = 0.931 for Ulcerative Colitis versus healthy individuals. On the UC–CD task, we observed a lower, yet relatively good, predictive performance (MCC = 0.688). This result might be due to the intrinsic difficulty in distinguishing between these two clinical subtypes of IBD, using solely colonoscopy-based diagnosis, whereas additional methods such as endoscopic ultrasonography may be more effective in differentiating UC from CD [25]. It should be noted that the results of the ensemble model demonstrate that there is only a marginal increase in predictive performance compared to using a single ResNet, possibly because it is difficult to further improve over already high-performance results. Overall, the obtained results in all the three classification tasks indicate a very good to excellent predictive performance, highlighting the potential of the framework to evolve into a valuable tool for the clinicians in their diagnostic tasks. Given the several subtleties of endoscopic imaging, linked to both the intrinsic disease features and to potential artifacts (for instance due to light effects), disagreement on the diagnosis is not uncommon among clinicians dealing with this task: the automated system proposed here can provide an additional view on the problem, clarifying the issues hampering the correct assessment of the pathology. Nonetheless, despite the encouraging results, the current study should be considered a proof of concept rather than a consolidated pipeline, which we have already planned to improve in future development. In this regard, additional model architectures other than ResNet could be evaluated, and different loss functions can be used to overcome the data limitation involving unbalanced classes. Moreover, the neural networks could be trained for more epochs, with additional combinations of hyperparameters, including automated approaches to data preprocessing and artefact removal, such as traditional image segmentation [26]. Additionally, alternative ensemble models can be tested, trained with images in different colour spaces. The model outcome can also be improved by enhancing the training data, even through augmenting techniques creating synthetic data: for example, generative adversarial networks. Finally, strengthening the resampling strategy will further improve the overall reproducibility of the study, while the analysis of the data trajectories across the DL layers can provide a valuable direction regarding the model interpretability.

## Conclusions

The results presented in this study demonstrate the vast potential of deep neural networks in discriminating between pathological and negative samples as well as discriminating between IBD subgroups, namely Ulcerative Colitis and Crohn’s Disease. Further development of this work and other studies in this area in general have the potential to become powerful tools in the hands of clinicians, aiding diagnosis and the clinical decision process. However, additional studies would be required to evaluate the utility of machine-learning aided diagnosis of IBD in clinical practice.

## Data Availability

The data generated and analysed during the current study are not publicly available due to privacy restrictions and commercial confidentiality, however they can be made available in a de-identified manner upon reasonable request and an appropriate agreement, contacting the corresponding author.

## Abbreviations

IBD: Inflammatory bowel diseases
CD: Crohn’s disease
UC: Ulcerative colitis
AI: Artificial intelligence
XAI: Explainable AI
DL: Deep learning
ML: Machine learning
RGB: Red, green, blue
HSV: Hue, saturation, value
ReLU: Rectified linear unit
ResNet: Residual neural network
MCC: Matthew’s correlation coefficient
TPR: True positive rate
TNR: True negative rate
PPV: Positive predictive value
NPV: Negative predictive value

## Footnotes

1 https://github.com/ufoym/imbalanced-dataset-sampler.

2 https://pytorch.org/hub/.

3 https://captum.ai/.
